# Using network analysis to elucidate the relationships among support systems, trauma and depressive symptoms, self-silencing, and risk of HIV viral non-suppression among black women living with HIV

**DOI:** 10.1007/s10865-024-00530-1

**Published:** 2024-11-23

**Authors:** Devina J. Boga, Reyanna St Juste, Kayla Etienne, Sannisha K. Dale

**Affiliations:** Department of Psychology, University of Miami, 5665 Ponce de Leon Blvd, Coral Gables, FL 33146 USA

**Keywords:** Black women, HIV, Social support, Viral non-suppression, Mental health

## Abstract

Human immunodeficiency virus (HIV) remains a major public health issue in the United States (US) and Black women living with HIV (BWLWH) are disproportionately impacted among women. This study investigates the complexities in influences of family, friend, and special person support systems and their association with post-traumatic stress disorder symptoms (PTSD), depressive symptoms, gendered coping (self-silencing), and a composite HIV risk score related to risk of viral non-suppression through missed medical visits, low medication adherence, and high viral load. Cross-sectional data among BWLWH were analyzed using network analyses via RStudio. Data from 119 BWLWH was reduced to 104, because of missing data on indicators as well as pairwise deletion for the correlation function. Findings revealed variances based on the type of network. For composite risk scores, friend support source had a weak to moderate significant correlation, while symptoms of PTSD and depression only showed a weak positive correlation with the composite risk variable through self-silencing as a form of coping. The post-hoc analysis showed a strong correlation with care as self-sacrifice, based on the composite risk score. Based on the findings from this study, insight was given into symptoms for depression and PTSD, as well as self-silencing and viral non-suppression risk in relation to sources of support for BWLWH. Future interventions to improve the overall health of BWLWH may benefit from incorporating support from friends and lowering care as self-sacrifice.

## Introduction

With new advents in medicine and healthcare, individuals living with HIV can live long and fulfilling lives, however life expectancy and quality of life are not equitable across race and gender. Factors such as systemic racism, discrimination, and financial marginalization are key drivers for the inordinate impacts of HIV among minoritized individuals including Black women (Earnshaw et al., 2013). New HIV diagnoses continue to disproportionately affect Black women and statistics from 2019 revealed that Black women exceed 50% of HIV incidence in women in the United States (US) (Centers for Disease Control and Prevention, 2020). However, after diagnosis follow up care is very important and may necessitate regular/frequent visits to providers for care and require adhering to a medication regiment (NIH, 2021) HIV Treatment Adherence. With the chronicity of HIV, it is important to understand what factors may ameliorate well-being among Black women living with HIV (BWLWH) in the face of the many challenges and barriers they face.

Studies have emphasized the importance of social support in health outcomes and well-being for BWLWH. Social support reflects the quality of relationships an individual has with others. One qualitative study found that emotional and informational types of support were important to BWLWH, particularly from other women living with HIV and from healthcare providers, especially around medication adherence (Koch et al., 2022). Another qualitative study highlighted how resilience was furthered by their loved ones including children, friends, family, peers, etc., and the ways that each source of support differed for those women in the level of perceived support. Sometimes support looked like childcare, gentle reminders to take medications, and an open space to be heard in non-judgmental way Dale & Safren, 2018). An egocentric social network analysis found that financial support was associated with lower depressive symptoms in a diverse group of racially/ethnically minoritized women and that having a family member among their network and/or receiving emotional support increased their general social support and treatment for HIV social support (Messer et al., 2020). When we parse out the sources of support in relation to disclosure, another social network analysis found that family members were disclosed to sooner than friend groups and in the longer term a larger proportion of friends would be disclosed to, but the implications of disclosure on those relationships were not studied (Serovich et al., 2012). However, there is a gap in the literature looking at the sources of social support and their relationship to multiple mental health outcomes along with HIV-related self-care focused on BWLWH who are statistically facing some of the most HIV related inequities.

There are elevated rates of mental health struggles among women living with HIV (Bing et al., 2001) with a longitudinal study among women indicating that the lifetime and one year prevalence and comorbidity of anxiety, mood and substance use disorders in women living with HIV exceeded that of the general population (Cook et al., 2018). Mental health issues can arise from factors including intersectional adversities (e.g., poverty, racism, sexism [(S. Dale et al., Under Review)]) trauma/violence exposure, and financial strains associated with HIV (Chuah et al., 2017; Remien et al., 2019). Additionally, mental health struggles may place women at risk for contracting HIV and suboptimal outcomes (e.g., adherence, engagement in care) along the HIV treatment cascade (Chuah et al., 2017; Remien et al., 2019). Interestingly, there is neurobiological pathway literature in which social support has been found to reduce the risk for developing mental illness, as well as epidemiological studies that show that low social support is associated with onset or relapse of depression (Ozbay et al., 2007). What is not yet clear is the specificity on which sources of social support may be more strongly related to the mentioned mental health outcomes.

In the context of relationships, the coping strategy of self-silencing has been associated with health outcomes such as depression and suicide (Jack & Dill, 1992; Maji & Dixit, 2019; Thompson & Dale, 2022). Self-silencing can be described as an individual silencing their feelings, thoughts, and actions in order to maintain interpersonal relationships. Specifically among women living with HIV self-silencing has been associated with medication nonadherence, increased viral load, and lower quality of life (Brody et al., 2014; Brody et al., 2014a). Another study focused on BWLWH found that substance use predicted lower rates of medication adherence and that this relationship was moderated by perceived social support, particularly friendship support (Reid & Dale, 2022). However, there is still limited information pertaining to sources of social support and health outcomes specific to BWLWH which is important to understand as the roles of women in these networks differ than those of men (Dale & Safren, 2018; Heckman et al., 2000; Lincoln et al., 2005).

Network analyses methods have been very illustrative and enlightening in the multiple fields of research such as, psychology, allowing researchers to understand the connections between various elements of syndromes that could better explain mental health processes, mediators, and points of intervention (Bloch-Elkouby et al., 2020; Borsboom & Cramer, 2013; Robinaugh et al., 2016). In public health, expanding to levels of influence more distal to the individual with an intersectionality approach the network may depict how structural influences interact and impact health outcomes while taking multicollinearity into account (Crenshaw, 2017; Lee et al., 2023; McElroy et al., 2021). The aim of this study is to explore the intricacies in family, friend, and special person support systems and their association with post-traumatic stress disorder symptoms (PTSD), depressive symptoms, coping (self-silencing), and a composite score related to risk of viral non-suppression through missed medical visits, low medication adherence, and high viral load in BWLWH. The central aim of this study is to explore the network and understand the correlations among the sources of support, mental health, and HIV-risk for viral non-suppression indicators. Hypothesis 1 is that there will be weaker support networks (weaker significant ties between support nodes) and stronger correlations between depressive symptoms, self-silencing, PTSD symptoms with the at-risk composite score. A secondary hypothesis is that friend support will be strongly associated with self-silencing in BWLWH.

## Methods

### Participants and procedures

Data was analyzed from the baseline data of 119 participants that were partaking in a behavioral medicine pilot randomized controlled trial for an intervention that addressed intersectional stressors to improve medication adherence. Participants were recruited through posting flyers in a university hospital medical system, local clinics and organizations, and community-based advertisement between November 2017 and January 2019. A phone screen was conducted to determine eligibility for the in-person baseline visit based on the following criteria: (1) identifying as a cis-gender woman (2) Black and/or African American (3) English speaking (4) having a history of abuse or trauma (5) being at least 18 years of age (6) prescribed ART (antiretroviral) therapy for the past two months at a minimum, and (7) indicating the possibility of low ART adherence, detectable viral load within the past 12 months, and/or missed HIV-related medical visits in the past 12 months. Women who met the phone screen criteria were scheduled for an in-person baseline visit, during which they were guided through an informed consent process. Women had to be able to fully understand and complete the informed consent process and study procedures. Individuals who were experiencing significant untreated mental health issues that would interfere with participation, unwilling to provide informed consent, or were in behavioral treatment for ART adherence or trauma in the past 6 months were excluded from the study. The baseline assessment entailed self-report measures and blood draws, and women were given a pill adherence monitor to use over the course of two weeks. Participants were compensated $50 for completion of baseline assessments. Ethics approval was provided by the X (blind review) Institutional Review Board.

### Measures

#### Socio-demographics

A demographic measure that included questions pertaining to age, education level, religion, sexual orientation, relationship status, children, housing arrangement/composition, income, employment status, and two items (one check all that apply and one open-ended item) from the psychosocial snapshot that asked about the individuals social support system.

#### Social support

Extensively used to measure social support, the Multidimensional Scale of Perceived Social Support (MSPSS) (Zimet et al., 1988) consists of 12 items (minimum score = 1, maximum score = 7) that divide into factor groups depending on the source of support, such as, family, friends, special person (global α = 0.93) (Dambi et al., 2018). Convergent validity for this scale with Social Support Behaviors scale has been found with high reliability in African/American and/or Black samples (Bronder et al., 2014).

#### Depressive symptoms

The Center for Epidemiologic Studies Depression Scale (CES-D) (Radloff, 1977) consists of 20 items that assess current affective depressive symptoms that participants experience in the past week. Sample items include “I felt that everything I did was an effort” and “I was bothered by things that usually don’t bother me”. This measure has been well validated across many populations including women living with HIV (Kalichman et al., 2000; Williams et al., 2007). The internal consistency in this sample was α = 0.88.

#### Coping through self-silencing

The Silencing the Self Scale (STSS) consists of 31 items that measures the way that individuals use coping through silencing/suppressing their thoughts, feelings, and behaviors in intimate relationships and in general to maintain relationships and minimize conflicts and/or discomfort for others (Jack & Dill, 1992). The following are the four subscales with sample items from each subscale: Externalized Self-Perception (6 items)- *“I tend to judge myself by how I think other people see me”*, Care as Self-Sacrifice (9 items)- *“Considering my needs to be as important as those of the people I love is selfish”*, Silencing the Self (9 items)- *“Instead of risking confrontations in close relationships*,* I would rather not rock the boat*, and Divided Self (7 items) - *“In order for my partner to love me*,* I cannot reveal certain things about myself to him/her”.* Responses are selected from a 5-point Likert scale of strongly disagree to strongly agree, with higher sum scores indicating more self-silencing. The internal consistency in this sample was α =.90, and the STSS has been validated among women living with HIV and Black women (Avery et al., 2022; DeMarco et al., 2001).

#### Post-traumatic stress disorder symptoms

Post-traumatic stress disorder (PTSD) symptoms were captured by the Davidson Trauma Scale (DTS), which is comprised of 17 items. For this analysis the frequency of symptoms was scored, and the responses were garnered through clinician interviews as opposed to self-report. The Cronbach’s alpha in the current sample was 0.84, the DTS has been validated across a variety of samples with different trauma exposures (e.g., childhood sexual abuse, war, natural disaster) (Davidson et al., 2002; McDonald et al., 2009; Zlotnick et al., 1996).

#### Composite risk score

Three items from the inclusion criteria were summed to indicate the risk score for viral non-suppression. A binary item was created for each of the indicators and they were later summed up for a total risk score that included (a) taking ART medication with low adherence (dichotomized cutoff of < 80%) OR (b) had a detectable viral load within the past 12 months OR (c) was at risk for becoming detectable as determined through missing at least one HIV-related medical visits in the past 12 months.

### Statistical analysis

Descriptive and network analyses were conducted using SPSSv28.0 (*IBM SPSS Statistics for Windows Version 28*, 2021) and RStudio version 2023.03.0 + 386 “Cherry Blossom” (*R: A Language and Environment for Statistical Computing*, 2020), respectively. Sociodemographic data was analyzed using means, standard deviations for continuous variables and frequency and percentages for categorical variables to describe the sample. Network analysis is effective in depicting how different determinants of health exist and interact in reciprocal symptom relationships through graphical visualization. The *qgraph (version 1.9.4)* (Epskamp et al., 2012) package was used with seven variables which were considered nodes denoted by circles in the visualization, and the edges represented relationships which were the ties (shown as lines) between the nodes. The relationships between nodes are further explained by looking at the thickness and distance of edges, the blue edges represented positive partial correlations and the red edges represented negative partial correlations. Importance of a node is often indicated by the number of edges or connections that it has to other nodes in the model, this is referred to as centrality (Smith et al., 2018). There are three main indices of centrality: node strength, closeness and betweenness. Higher values for all three indices imply the node is more important. Node strength is derived through the sum of absolute edge weights connected to the nodes, betweenness is calculated by looking at the frequency of how often a node is in the shortest path between other nodes and closeness is quantified by taking the inverse of the total of distances from one node to remaining nodes within the network (Epskamp & Fried, 2018). A fourth indicator of centrality is expected influence, this indicator accounts for the negative edges found in the network improving the assessment of influential nodes by looking at the cumulative influence (Robinaugh et al., 2016). The nature of the data lends to utilization of the *cor_auto* function to obtain a partial correlation network model that controls for other relationships among the variables and is estimated using lasso regularization techniques, this function accounts for combinations of data that could be ordinal (polychoric), continuous(Pearson) or both (polyserial) (Epskamp & Fried, 2018). This regularization allows for the model to be simplified for interpretation by removing spurious edges through a penalty for model complexity, allowing for both model-selection and parameter estimation (Epskamp & Fried, 2018). The Extended Bayesian Information Criteria (eBIC) hyperparameter was set to γ = 0.5 to represent a parsimonious and sensitive network (Epskamp & Fried, 2018). We will specifically look at the interconnected relationship among three sources of support (friends, family, and special person) and their association with depressive symptoms, Post-Traumatic Stress Disorder (PTSD) symptoms, coping and a composite at-risk score of viral non-suppression.

## Results

A total of 119 women completed baseline measures, however due to missingness on indicators and pairwise deletion in the correlation function, 104 women were included in the analyses. As shown in Table 1, the mean age was 50.4 years with a standard deviation of 10.1 years. Many of the women had some high school education (29.8%), a high school diploma or GED (31.7%), and some college education (26.9%). A large proportion of the women were either not working or on disability (81.7%) and reported an annual income of less than $5,000 (34.6%). Almost 70% of the women were currently renting a home or apartment. Most women were single (45.2%) followed by not married and living with a partner (15.4%) and married (13.5%) and more than half of the women identified as exclusively heterosexual (77.9%). A majority of the women had children (82.7%) and practiced the Baptist religion (53.8%). In the psychosocial snapshot, women were asked to check all options that applied regarding social support with a variety of options as well as an open-ended question that elaborated on social support that may not have been captured by the item. From most endorsed to least endorsed source of support findings were children (count = 48), friends (47), professionals (32), siblings (33), partners (31), parents (19), peers (12), and lastly grandchildren (5). In response to the open-ended item, the support of God was mentioned by 17 participants. The average PTSD symptom score was 43.3 (SD = 27.2) in this sample, indicating a high level of trauma symptoms (Tang et al., 2020). Similarly, a score of 16 or greater on the 20-item CES-D is indication of probable clinical depression and in the current sample the mean depressive symptom score was 22.2 (SD = 11.4) (Vilagut et al., 2016). The average STSS total was 83.0 (SD = 24.9), with the range of 12 to 137. Results for the perceived social support sources were as follows: average score for family support was 4.69 (SD = 1.8), the mean friend support was 4.71 (SD = 1.6), and average score for special person support was 5.34 (SD = 1.4). The minimum number of criteria for the composite risk score variable was 0 and the maximum was 3, meaning an individual had met all 3 criteria for increased risk of viral non-suppression. The counts in our sample for each level were 57 individuals met 0 criteria, 40 individuals met 1 criterion, 12 individuals met 2 criteria, 3 individuals met all three criteria, and 7 individuals were missing data on the composite risk score variable.

**Table 1:** Sociodemographic characteristics (*n* = 104)

Characteristics	Mean (SD) / *N* (%)
Age Mean (Standard Deviation)	50.4(10.1)
Education n (col%)	
≤8th grade	4(3.8)
Some HS	31(29.8)
HS graduate/GED	33(31.7)
Some college	28(26.9)
College graduate	7(6.7)
Some graduate school	1(1.0)
Relationship status	
Married	14(13.5)
Not married, living with partner	16(15.4)
Non-cohabitating relationship	12(11.5)
Single	47(45.2)
Divorced/separated	10(9.6)
Loss of long-term partner/widowed	3(2.9)
Missing	2(1.9)
Sexual Orientation	
Exclusively heterosexual	81(77.9)
Heterosexual with some same-gender loving experience	9(8.7)
Bisexual	6(5.8)
Gay with some heterosexual experience	−
Exclusively gay	3(2.9)
Choose not to answer	5(4.8)
Religion	
Christian	28(26.9)
Catholic	4(3.8)
Baptist	56(53.8)
Other	7(6.7)
None	9(8.7)
Household characteristics	
Children Yes No Missing	86(82.7) 18(17.3) 1(0.8)
Employment Status	
Full time or Part time work	11(10.5)
Full time or Part time in School	5(4.8)
Neither work nor school/Disability	85(81.7)
Other/choose not to answer	7(6.7)
Income	
< 5,000	36(34.6)
5,000 ≤ x ≤ 11,999	29(27.9)
12,000 ≤ x ≤ 15,999	8(7.7)
16,000 ≤ x ≤ 24,999	5(4.8)
25,000 ≤ x ≤ 34,999	3(2.9)
35,000 ≤ x ≤ 49,999	2(1.9)
≥ 50,000	3(2.9)
Missing/refuse to answer/don’t know	18(17.3)
Housing	
Renting home or apartment	72(69.2)
Own apartment or home (self/someone else in household)	12(11.5)
Publicly subsidized housing	11(10.6)
A friend/relatives’ home (pay little or no rent)	6(5.8)
Unhoused (shelter, street, car, etc.)	2(2.0)
Social Support	
Children	48(46.2)
Grandchildren	5(4.8)
Siblings	33(31.7)
Parents	19(18.3)
Friends	47(45.2)
Partners	31(29.8)
Peers	12(11.5)
Professional	32(30.8)

**Fig. 1:**
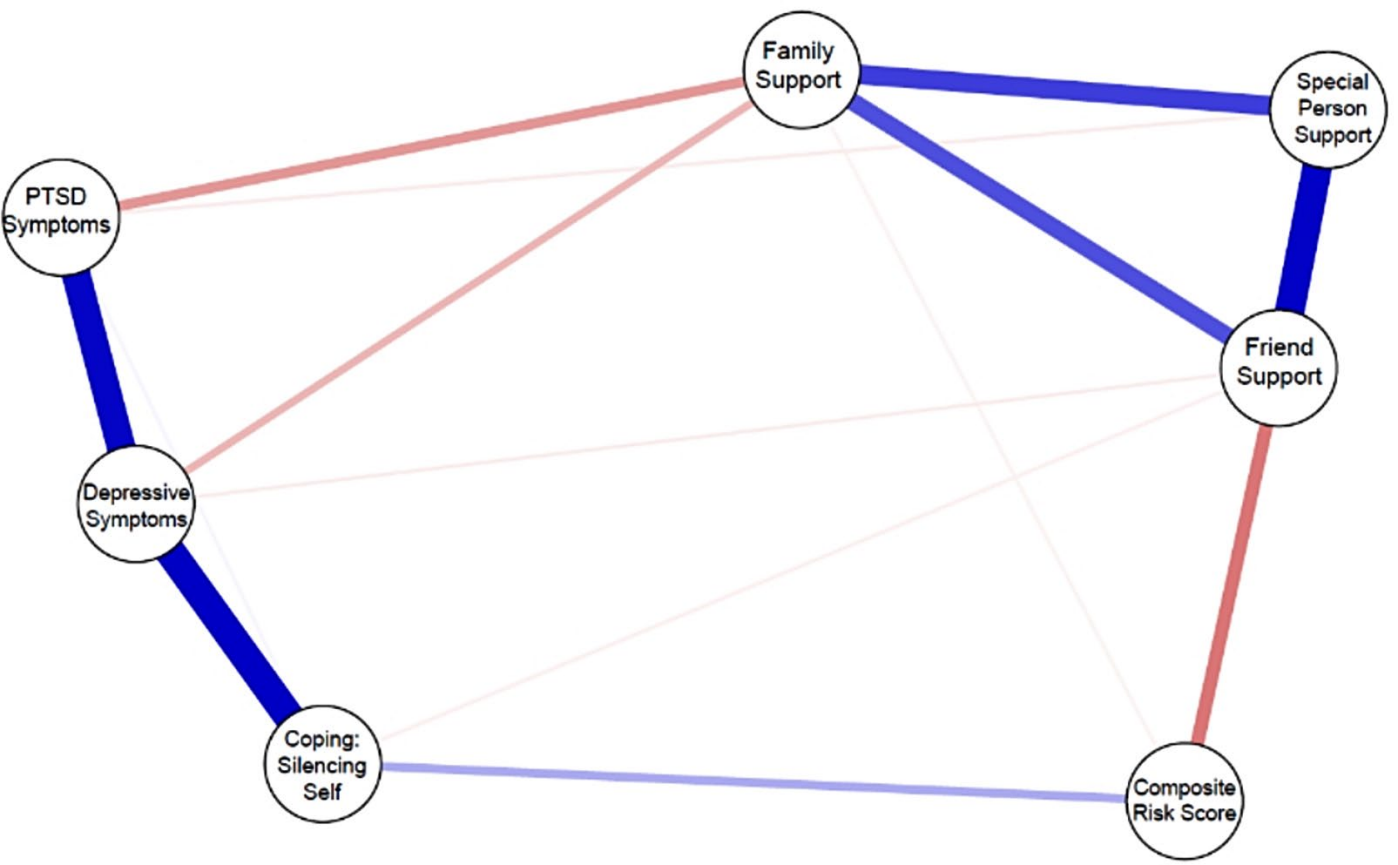
Network of sources of support, mental health, coping and risk for viral non-suppression indicators

The network analysis results showed an undirected network with weighted edges portraying the presence and strength of both positive and negative connections amongst our variables of interest (see Table 2 for the partial correlation matrix). As depicted in Fig. 1, the blue edges in the network show stronger positive partial correlations in three pairwise relationships after controlling for all other variables: friend and special person sources of support (*r* = .451), coping through self-silencing and depressive symptoms (*r* = .464), and PTSD symptoms and depressive symptoms (*r* = .437). Moderate positive partial correlations relative to the network were found in two pairwise relationships: between friend and family support sources (*r* = .254) and between family and special person support sources (*r* = .290) and a weaker positive correlation was found between coping through self-silencing and the composite risk variable (*r* = .110). Moderate to weak negative connections were found between friend support and composite risk score (*r* = − .190), and between family support and PTSD symptoms (*r* = − .137) and family support and depressive symptoms (*r* = − .095). The weakest negative connections were found between special person support and PTSD symptoms (*r* = − .022), family support and the composite risk score (*r* = − .013), and friend support with depressive symptoms (*r* = − .021) and with coping through self-silencing (*r* = − .017).

**Table 2:** Partial correlation matrix between indicators

	CES-D	PTSD	Silencing Self	Composite Risk Score	Special Person Support	Family Support	Friend Support
CES-D	−	−	−	−	−	−	−
PTSD	0.436	−	−	−	−	−	−
Silencing-Self	0.464	0.009	−	−	−	−	−
Composite Risk Score	0.000	0.000	0.110	−	−	−	−
Special Person Support	0.000	− 0.022	0.000	0.000	−	−	−
Family Support	− 0.095	− 0.137	0.000	− 0.132	0.290	−	
Friend Support	− 0.021	0.000	− 0.171	− 0.190	0.452	0.254	−

**Fig. 2:**
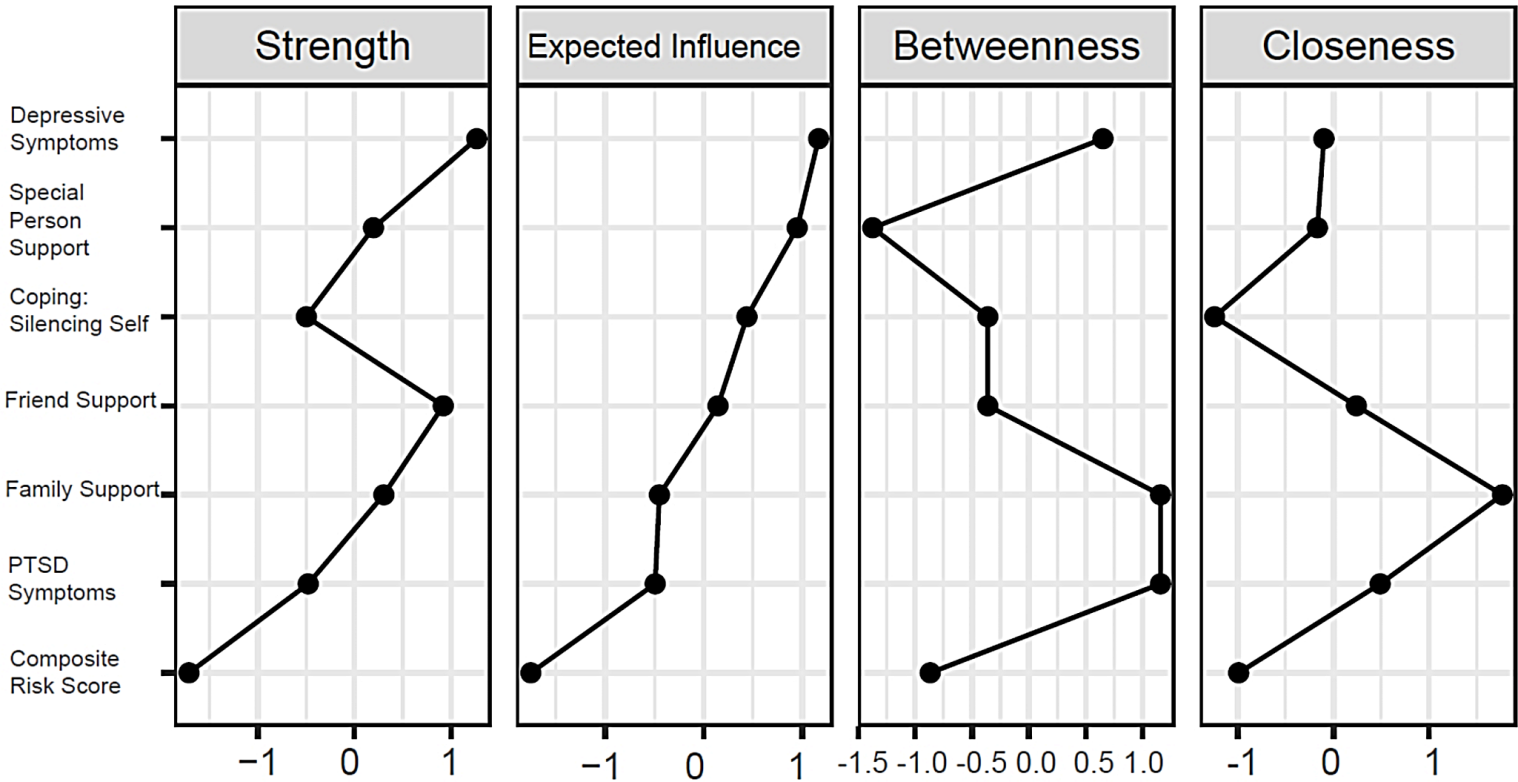
Centrality indices

The standardized z-scores for the four centrality indices are displayed in Fig. 2. The node for depressive symptoms displayed the highest strength (r_s_ = 1.02), expected influence (r_ei_ = 0.78), and high betweenness (r_b_ = 4) on/with other nodes in the network. Both family support and PTSD symptoms exhibited the highest betweenness (r_b_ = 5), meaning they served as bridges between other nodes in the network, family support also had the highest closeness (r_c_ = 0.22). The lowest and second lowest centrality on all indices was exhibited by the composite risk score (r_s_ = 0.31, r_ei_ = − 0.09, r_b_ = 1, r_c_ = 0.18). The three support sources showed a clustering of strong positive correlations among each other, we did not see the same clustering pattern with the mental health symptoms (PTSD symptoms, depressive symptoms and coping through self-silencing). As hypothesized, friend support was associated with self-silencing, however it was a weak correlation.

## Post-hoc analysis

**Fig. 3:**
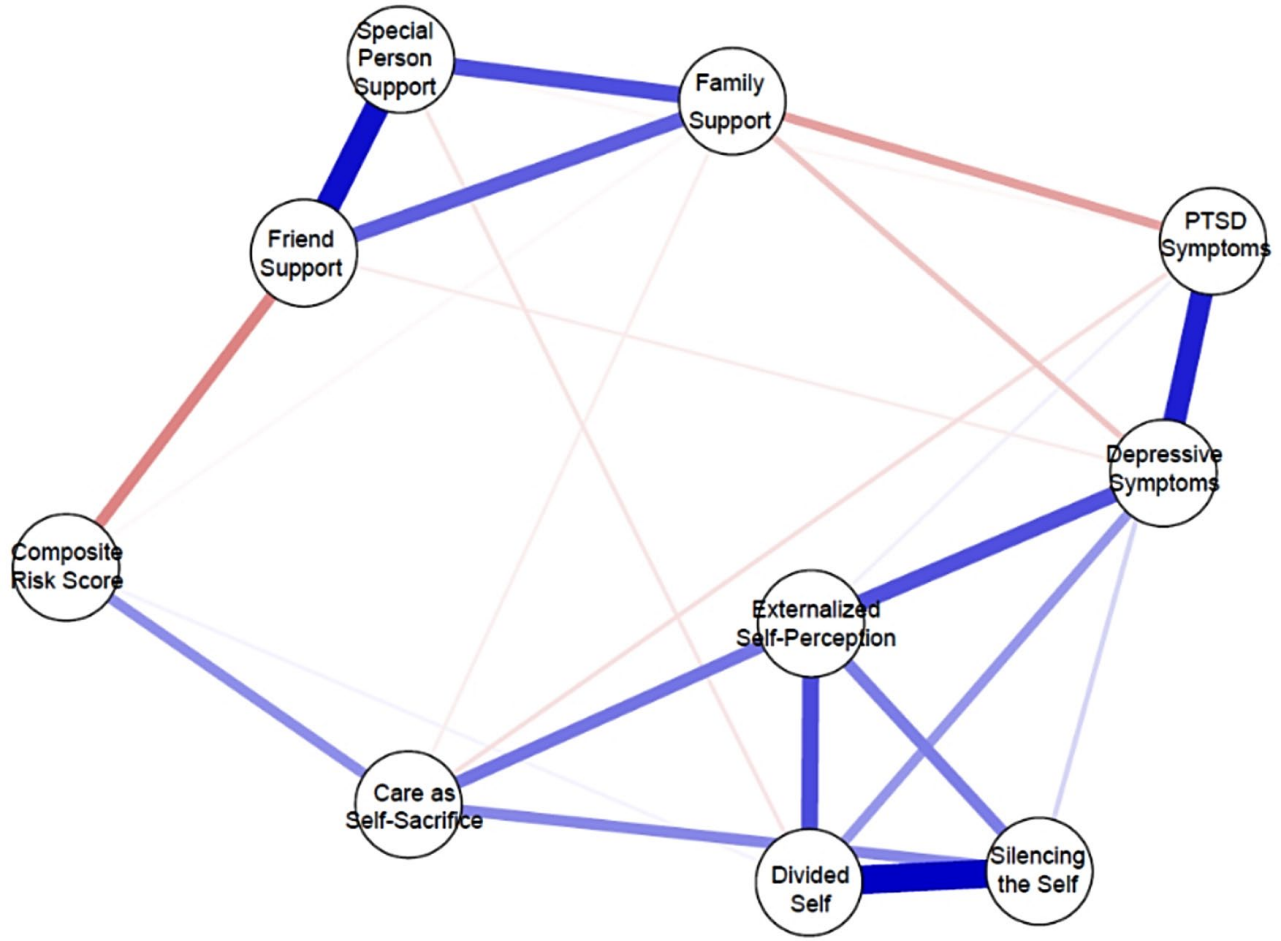
Network with subscales of the silencing the self scale

An exploratory approach was taken to further understand the relationship found between our hypothesized relationships. Coping through self-silencing was the only indicator amongst the mental health indicators that was associated with the composite risk score. Thus, a secondary network was estimated with the subscales of the STSS measure instead of the total measure score to gain more understanding of what subscales of the STSS may be driving the relationships. As presented in Fig. 3, the Care as self-sacrifice subscale was correlated to the composite risk score after controlling for all other variables in the network (*r* = .17), the Divide-self subscale was also weakly associated with composite risk (*r* = .01). In the separation of the STSS measure, the correlation of friend support was not retained, however family support was weakly associated with the care as self-sacrifice subscale (*r* = − .021). Table 3 shows the weighted partial correlation matrix of the network with the STSS subscales.

**Table 3:** Partial correlation matrix between in post-hoc analysis indicators (STSS subscales)

	CES-D	PTSD	Composite Risk Score	Special Person Support	Family Support	Friend Support	Ext Self-Perception*	Care As Self-Sacrifice	Silencing the Self	Divided Self
CES-D	−	−	−	−	−	−	−	−	−	−
PTSD	0.397	−	−	−	−	−	−	−	−	−
Composite Risk Score	0.000	0.000	−	−	−	−	−	−	−	−
Special Person Support	0.000	− 0.010	0.000	−	−	−	−	−	−	−
Family Support	− 0.082	− 0.140	− 0.008	0.284	−	−	−	−	−	−
Friend Support	− 0.022	0.000	− 0.185	0.446	0.255	−	−	−	−	−
Ext Self-Perception*	0.281	0.019	0.000	0.000	0.000	0.000	−	−	−	−
Care As Self-Sacrifice	0.000	− 0.043	0.168	0.000	− 0.021	0.000	0.215	−	−	−
Silencing The Self	0.000	0.059	0.000	0.000	0.000	0.000	0.198	0.182	−	−
Divided Self	0.155	0.000	0.014	− 0.034	0.000	0.000	0.292	0.000	0.519	−

* Externalized Self Perception

## Discussion

Among our sample of BWLWH we display the relationships between sources of support, mental health symptoms, coping, and risk for viral non-suppression through a network analysis. The first research question in the study sought to understand the ties between the support networks and the mental health symptoms/coping and the composite risk score. Findings revealed that the three support subscales were strongly associated with one another, although there was a closer and stronger tie between friend support and special person support. The friend support source was the only support scale to have a medium significant correlation to the composite risk of viral non-suppression. More importantly the relationship was negative in nature, suggesting that friendships play an important role in HIV-related adherence and self-care for the women in the sample. Historically there has been a lack of peer-led interventions that focused on BWLWH, The Black Women First Initiative (2020–2024) was implemented in twelve demonstration sites where multileveled bundled interventions were offered to address use of HIV care services, continued engagement in care, and social determinants of health for the wellbeing of BWLWH. Nine out of twelve sites included an enhanced peer/patient navigator component, typically the peers in these interventions are also living with HIV. In our study, friend support was not exclusive to those sharing status, indicating that interventions maybe expanded to include support from established friends in a woman’s support circle. In assessing the relationship between mental health symptoms and the composite viral non-suppression risk score, we found depressive symptoms and PTSD symptoms were only related to ( the composite risk score by way of coping by self-silencing (Rajabiun et al., 2023). Our results support what has been found in the literature. Strong associations between self-silencing and HIV related prevention or care have been found amongst college-aged Black women and BWLWH specifically (Brody et al., 2014a; Lanier & DeMarco, 2015; Stokes & Brody, 2019). Future public health and clinical interventions aimed at improving HIV related care should take this into consideration and aim to harness the strengths within a support network for BWLWH while lowering self-silencing to improve HIV related self-care. Routine questionnaires in clinical settings and points of care for BWLWH may include brief items on mental health, self-silencing, and social support as a first step to build awareness for an individual through a self-reflective process and an opportunity for providers to ask applicable questions and appropriate referrals to resources (e.g., support groups, therapy).

The ties between the support networks and mental health symptoms/coping could be explained by the overlap that may exist in understanding/delineation of special person versus a friend, it should be noted that almost half the women were single in this study. Previous studies have reported a unifactorial or a two-factor structure of the MSPSS, where the special person subscale joined into the friend or family scales (Dambi et al., 2018; Kim et al., 2022; Stanley et al., 1998). A prior research study has also found that friends were perceived as more supportive than family members for a group of men and women living with HIV (Brody et al., 2014a). The current study highlights for which health outcomes/behaviors support is especially pertinent. Within the same network, out of the support sources we found that family support was most strongly negatively correlated with both depressive symptoms and PTSD symptoms. It is possible that different types and sources of support are more salient for different needs/outcomes. Another study that assessed social support in relation with medication adherence among men and women living with HIV looked at support through any source and the type of support (appraisal, emotional, spiritual), however our study gives insight into which source of support may be helpful for intervention (Simoni et al., 2006). Depressive symptoms having the highest centrality or importance in the first network is consistent with literature indicating that depression was one of the most common psychiatric comorbidities in people living with HIV (Nedelcovych et al., 2017). Our finding that depressive symptoms and PTSD symptoms was only related to the composite risk score by way of self-silencing is novel, but also aligned with prior literature linking self-silencing with depressive symptoms and HIV outcomes among women living with HIV (Brody et al., 2014a; Shahid & Dale, 2024).

The second research hypothesis was that friend support would be related to self-silencing. We initially found that friend support was related to coping through self-silencing, however when we looked at the subscales of the STSS this relationship was not retained. Instead, we saw that special person and family support were related to the constructs of divided-self and care as self-sacrifice, respectively. The divided-self subscale items really dive into the relationship between the individual and their partner, in a previous study the divided-self subscale was found to mediate the relationship between abuse and ART adherence in Black women (Brody et al., 2014a). A previous study has found a correlation between trauma and STSS in Black women (Harrington et al., 2010), however it was not replicated in our findings of the initial network looking at PTSD symptoms as a measure of trauma. In the post-hoc network, the care as self-sacrifice subscale was negatively associated with PTSD symptoms and family support. The burden of maintaining a household, understanding and meeting the needs of others often falls in the purview of women (Elliott et al., 2015). The connection for family support through care as self-sacrifice to the composite risk score visualizes this experience in our sample. Family support can be bidirectional, as there is increased support through family women may feel the need to show they care through self-sacrifice which may look like sacrifices of time (not making it to appointments) and finances (not being able to get medical needs met) which impact their health-related self-care. Authors Morris and Kahlor (Morris & Kahlor, 2018) used a discourse analysis to understand the implications of individual responsibilities messaging used in HIV-related public service announcements and they found that the current messaging perpetuates the historical burden imposed on Black women to further their community through self-sacrifice for family and unconditional support to their partners. Post-hoc analysis results revealed that the composite risk score for viral non-suppression was most strongly correlated with the care as self-sacrifice subscale of STSS.

## Limitations

The current study is limited by a few factors. The relatively small sample of 119 participants was reduced to 104 participants as pairwise deletion methods are used in partial correlation estimation. Another limitation is related to the measure of social support used in our study; it should be noted that the type of support emphasizes emotional support rather than instrumental support. This study used a cross-sectional design; hence relationships were estimated through associations as causality and temporality cannot be inferred. The generalizability of these results is subject to limitation as the sample was limited to Black women living with HIV and histories of trauma in the Southeast U.S.

## Conclusion

This study offers interesting insights into the sources of support and their associations with depressive symptoms, PTSD symptoms, coping through self-silencing and risk for viral non-suppression in BWLWH. Using network analyses methods this study helped to identify the characteristics of support networks and their associations with important health behaviors and health outcomes in BWLWH that can potentially inform programs or interventions that could utilize or harness the power of interpersonal support systems. Future studies should further study the ways in which friend support may help women with HIV-health self-care and coping outcomes. Public health interventions at the societal and system level need to be in place, where child and family support systems are more readily available and affordable so that women can focus on their own well-being. Also harnessing family and special person support in an intervention may help to decrease depressive and PTSD symptoms which are common mental health struggles among BWLWH.

## Data Availability

The details and findings of the larger clinical trial, under which these research findings are subsumed, are available for open access at clinicaltrials.gov (Trial Registration Number: NCT02764853; Date of Registration: May 3, 2016).

## Code availability

N/A.
